# Development and Validation of the Body Cognition Assessment System

**DOI:** 10.3390/brainsci13091237

**Published:** 2023-08-24

**Authors:** Ikumi Ikejiri, Takashi Murakami, Ryosuke Yamauchi, Hideaki Yamaguchi, Takayuki Kodama

**Affiliations:** 1 Graduate School of Health Sciences, Kyoto Tachibana University, Kyoto 607-8175, Japan; h901121004@st.tachibana-u.ac.jp (I.I.); h901522007@st.tachibana-u.ac.jp (R.Y.); 2 Kyoto Tachibana University, Kyoto 607-8175, Japan; murakami-tak@tachibana-u.ac.jp (T.M.); caretech.plus.hy@gmail.com (H.Y.); 3 Department of Rehabilitation, Kyoto Hakauikai Hospital, Kyoto 603-8041, Japan; 4 CARETECH plus, Nagoya 462-0847, Japan

**Keywords:** BCAS, body cognition, FBT

## Abstract

Body awareness, which comprises the sense of body possession and action ownership, is essential for the adaptive movement of humans in response to external environments. However, existing body cognition assessments include many overt elements of cognitive functional activity, but no assessment captures the latent body cognition necessary for exercise and daily life activities. Therefore, this study aimed to devise a body cognition assessment system (BCAS) to examine the functional basis of body cognition in healthy participants and investigate its usefulness. The BCAS was used to assess body cognition on three occasions, and BCAS values were calculated from the results of the assessment. The intraclass correlation coefficient (ICC) was used to determine reproducibility. Neural activity in the brain during somatocognition assessment while conducting the BCAS was measured by electroencephalogram. Moreover, the functional basis for somatocognition with the BCAS was also investigated. The results demonstrated that the BCAS values varied across the three administrations (ICC (1.3) = 0.372), and changes in the state of neural activity in the brain were observed. The results suggest that assessment using the BCAS may be a new indicator of ever-changing body cognition.

## 1. Introduction

In everyday life, humans possess appropriate body cognition based on various perceptual functions of the body that adapt to the external environment [1]. This is called “body perception” and consists of a sense of body ownership and action subjectivity. The sense of body ownership is the awareness of body possession that “this body is my body” [2], the creation of which is based on the activity of brain regions, such as the parietal association cortex, premotor cortex, and insular cortex. On the other hand, a sense of action ownership refers to the body-ownership consciousness that, “I am the one who realizes my movement” [2]. The sense of action is based on the activity of the supplementary motor, prefrontal, and parietal association cortices. Body cognition is constructed by the spatiotemporal match between the predicted sensory information generated by the intention of active movement and the actual sensory information; thus, movement adapted to the external environment is carried out [2]. Therefore, body awareness is indispensable for humans to adapt to the ever-changing external environment for survival. However, when the central nervous system sustains damage from neurological disorders such as stroke, sensorimotor dysfunction occurs, resulting in a discrepancy between sensory prediction and actual sensory information, and an alteration in body perception occurs [3].

Approximately 85% of patients with stroke experience some degree of paralysis in the upper limbs [4], and another 50% present with functional impairment of the upper limbs and fingers during the “chronic phase” [5]. The human upper limb is essential for daily activities such as eating, dressing, grooming, and bathing. Loss of upper limb function due to sensorimotor dysfunction following stroke interferes with the activities of daily living (ADLs) and is associated with a reduced quality of life (QOL). Loss of upper limb function is associated with a decline in ADL and QOL [6,7,8]; therefore, rehabilitation interventions for paralyzed upper limbs with sensorimotor dysfunction are important for improving the QOL of patients with stroke [7].

Improving physical function is essential to improve the QOL of patients with stroke; therefore, reconstructing the “body perception” of the paralyzed upper limb and increasing the frequency of its use in everyday life is essential [3]. Upper limb sensory-motor dysfunction is believed to cause an alteration in “body perception” [9]. Miyawaki et al. [10] have demonstrated that, due to sensory-motor dysfunction, stroke patients make false self/other attributions, judging others’ movements as their own. This indicates that stroke patients have difficulty distinguishing their movements and those of others, leading to altered body cognition, and the symptoms interfere with ADLs and lead to reduced QOL. Therefore, it is important to assess brain functional status to elucidate somatocognitive alterations in persons with sensory-motor dysfunction in stroke.

In body cognition evaluation, the sense of action subjectivity constitutes an aspect of the process of creating a sense of body ownership [11]. Therefore, in voluntary physical exercise, both the sense of action subjectivity and the sense of body ownership are mixed and expressed [12,13]. Thus, a compatibility perspective should be adopted to clearly understand body cognition [2,14,15]. Furthermore, Synofzik [16] reported body perception to possess a layered structure with latent (sensory) and manifest (cognitive) levels, both of which modify one another. At the latent level, nonconceptual body cognition is generated using comparator models in the brain, and it is constructed by the spatiotemporal match between the prediction of sensory information generated by active motor intentions and the actual sensory information in the comparator model. In the event of a discrepancy, a conceptualization system at the manifest level comes into play, utilizing intentions, thoughts, beliefs, and context to determine who owns the discrepancy and who is the acting subject. Healthy individuals perform bodily movements at a subconscious level daily, without being aware of their bodies. In patients with stroke, the paralyzed limb no longer produces movements commensurate with the amount of effort, resulting in a discrepancy between the predicted and actual sensory information, which is thought to cause an alteration in body cognition [3]. For example, in the rubber hand illusion [17], a representative experiment of body possession, when tactile stimuli are applied synchronously to real and rubber hands simultaneously while observing only the rubber hand, the discrepant stimuli are perceived as if it were the participant’s own. Therefore, multisensory integration of visual and somatosensory sensations in a spatiotemporally coincident state elicited a sense of actual bodily possession. Shimada et al. [18] reported that by creating a temporal delay in visual feedback, the sense of action ownership was significantly reduced owing to a temporal discrepancy between the prediction of sensory information and the actual sensory feedback. The findings suggest that improving ADLs and QOL requires building body cognition at a latent level while simultaneously capturing immediate changes in body cognition.

An intervention study [19] reconstructed the body cognition of the upper limb in patients with sensorimotor dysfunction using somatomotor imagery as the basis of body cognition. A Cochrane review [20] (2020) reported motor imagery therapy to be effective in improving upper limb motor function. Moreover, somatic motor imagery is a mental motor representation without physical movement that is reproduced by working memory, which produces brain activity similar to that of real movement, such as in the premotor and supplementary motor areas, parietal association areas, and prefrontal cortex, especially in the brain regions involved in the preparation and planning of movement [21]. However, whether patients with cerebrovascular disease and altered body perception of the paralyzed limb can accurately visualize the body and movement is still debated [22,23,24], and assessments capturing changes in body perception before and after the intervention have not yet been reported.

Thus, the method of assessing body perception, which instantly varies from day to day, is important for the rehabilitation of patients with sensorimotor dysfunction.

Considering previous assessments of body cognition, the forearm bisection test (FBT) [25,26,27], sense of agency task (Keio method) [28,29], numerical rating scale (NRS) [18], the Kinesthetic and Visual Imagery Questionnaire (KVIQ) [30,31], and the motor imagery questionnaire (MIQ) [32,33], which are paper-based assessments, have been reported. The FBT assesses body perception based on a static body image by pointing to the midpoint of one’s forearm on a desk and measuring the error between the actual midpoint and subjective midpoint [25]. The KVIQ and MIQ are objective assessments that evaluate somatomotor imagery of whole-body movement without actual movement. However, for the following existing assessments: FBT assessing body perception on the basis of static body imagery [25] and the NRS quantifying through a cognitive processing process [34] (which may be influenced by cognitive and higher brain functions), a persisting challenge may be the difficulty to capture body cognition mixed with a sense of body ownership and action ownership under a static task. In addition, the KVIQ and MIQ do not reflect the somatomotor cognitive abilities of the target limbs, which are impaired in patients with sensorimotor dysfunction. Therefore, they most likely are an indirect assessment at the manifest level and are unreliable, and their use as evaluation indexes with reliability is challenging [35,36]. In addition, the sense of agency task (Keio method) [29], which is an evaluation of the sense of action subjectivity, is an experiment in which the experience of the temporal causal linkage between the intentional action and the resulting external event is evaluated (using a computer and the time between the participant’s operation (button pressing) and the response on the screen). The bias was programmed in milliseconds, and the participant was asked to assess whether they felt that they were the subject of the action. Specifically, when the participant pressed a button on the keyboard, the target moving on the laptop jumped, and a time bias of 0–1000 ms was randomly programmed between the button press and the jump so that the participant could judge whether they had moved the target by themselves. This is an evaluation in which participants are asked to choose between “self” and “non-self” for the question if they felt that they moved the target. This requires evaluation of the sense of action subjectivity after manipulating only the fingertips. As it is not a direct evaluation of the original physical cognitive ability of the target limb, it tends to be an indirect evaluation at the apparent level and is not an evaluation index of potentiality [37]. Therefore, for example, in the case of the upper limb, a direct somatocognitive assessment at a potential level is required, with the assessment task being a movement that is representative of a movement disorder and relatively easy to visualize in the original movement of the upper limb function (e.g., raising the upper limb in the forward direction). However, these body cognition assessment methods have not yet been developed. Hence, we have developed a new body awareness assessment system to directly assess subconscious body awareness.

Therefore, the present study aimed to use our newly developed body cognition assessment system (BCAS) to correspond the results obtained with the established assessments (using the KVIQ and FBT) and brain function from a neurophysiological perspective. Additionally, this study sought to investigate the reliability and usefulness of the BCAS as a potential physical and cognitive assessment tool.

## 2. Materials and Methods

### 2.1. Participants

The participants were 16 healthy male students (mean age 21.2 ± 1.6 years) enrolled in the Department of Physiotherapy, Faculty of Health Science, Kyoto Tachibana University (Table 1). Handedness was assessed using the Edinburgh Handedness Inventory, and all participants were right-handed [38]. The exclusion criteria were visual impairment or orthopedic, neurological, or psychiatric disorders that would make it difficult to perform the study task. The results of the interview survey revealed that none of the participants could be excluded. Students from the authors’ university were selected after being verbally explained the content of the experiment. Furthermore, the participation of the students was voluntary. Upon meeting, the experimenter explained the purpose, methods, benefits, disadvantages, and risks of participation in the study, orally, and obtained consent in writing. The participants were also informed that they could refuse to answer the questions or participate in only some measurements. The study was approved by the Ethics Committee of Kyoto Tachibana University (approval number: 21–34).

### 2.2. Experimental Protocol

The study used a cross-sectional, within-subjects design. The experiment aimed to optimize the assessment of body cognition using comparative validation with the KVIQ and FBT to verify the usefulness of the BCAS. In addition, electroencephalogram (EEG) measurements were obtained during the assessment using the FBT to validate the areas of brain activity. The test was performed on the right upper limb using the KVIQ, a motor imagery recall assessment [30], and the FBT, an assessment of body possession [25,26,27]. The BCAS developed in the study was used for evaluation.

**Figure 1:**
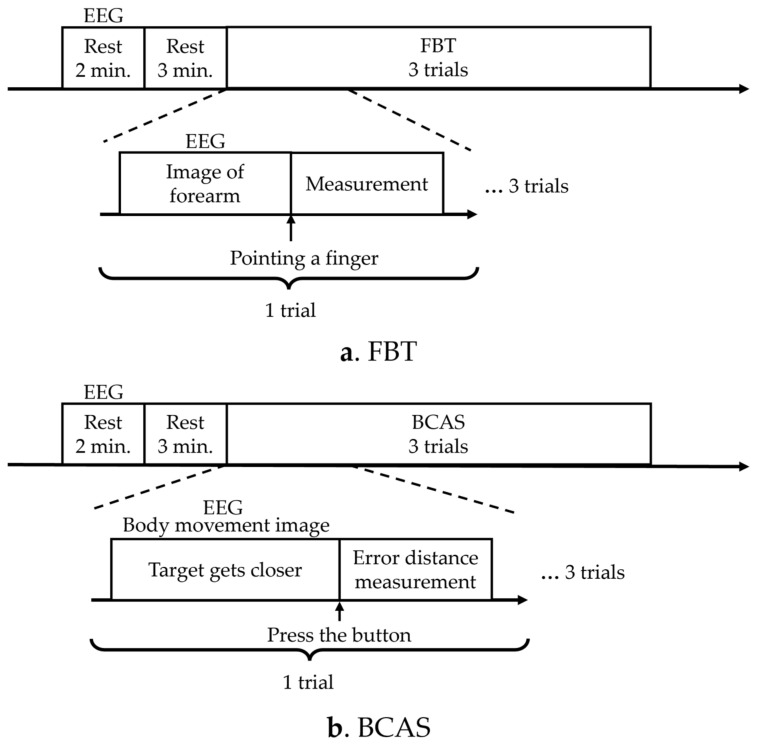
Experimental protocols. (**a**) The diagram illustrates the experimental protocol for FBT. (**b**) The diagram presents the experimental protocol for BCAS. EEG: electroencephalogram; FBT: forearm bisection test; BCAS: body cognition assessment system.

The KVIQ was measured before the FBT and BCAS experiments, as a control, to detect the body’s perception of visual information immediately before the experiments. The sequence of the FBT and BCAS experiments was repeated three times in a different order for each participant to account for order effects, and the EEG measurements were obtained to compare brain activity during each assessment (Figure 1). The number of times the FBT and BCAS were administered was set with reference to the number of times used in the previous motor imagery study [39], while considering that the brain’s motor learning was updated by administering them multiple times.

In the FBT, the resting EEG signal was first measured for 2 min, followed by a resting state for 3 min. In FBT, the participant pointed to the secondary subjective midpoint of the right forearm upon instruction to start pointing, and the distance from the elbow head to the midpoint point was measured once, which was performed three times. EEG measurements were performed simultaneously and recorded from the instruction to start pointing, until the participant pointed to the subjective midpoint of the right forearm (Figure 1a). In the BCAS, resting EEG was measured for 2 min, followed by 3 min of rest. The target then began to approach from the front. The subject pressed a button to stop the target, and the error between the predicted and actual arrival points was measured. The distance measurement was performed once and three times. EEG measurements were performed simultaneously from the moment the target started approaching the participant until the participant pressed the switch to stop the target (Figure 1b). A washout period of 5 min was allowed between the assessments.

### 2.3. Assessment of Motor Imagery Recall (KVIQ)

To investigate the correlation between our newly developed BCAS and established motor imagery assessments, we used the FBT and KVIQ, a motor imagery recall assessment questionnaire that measures how well a person can see or feel an imagined movement [30]. Participants were asked to imagine the movement from a first-person or internal perspective (as if they were performing the movement themselves) in a seated position. The questionnaire included a visual and muscular sensory imagery scale. As subjective imagery was the aim of the present study, however, only the more subjective muscular sensory imagery scale was measured. Furthermore, for the body perception tool, only the “3Knd Forward shoulder flexion”, “4Kd Elbow flexion/extension”, and “5Kd Thumb-finger-opposition” information were extracted and incorporated into the KVIQ to assess body perception, from the shoulder joint to the tip of the hand, when the upper limb was extended forward. On the Muscle Sensory Imagery Scale, the participant was asked to evaluate the imagery themselves, with “strong as if performing the movement” as 5, “strong as 4”, “moderately strong” as 3, “slightly strong” as 2, and “no sensation” as 1. The participants with high scores on this question were judged to possess high motor image recall.

### 2.4. Forearm Bisection Task (FBT)

**Figure 2:**
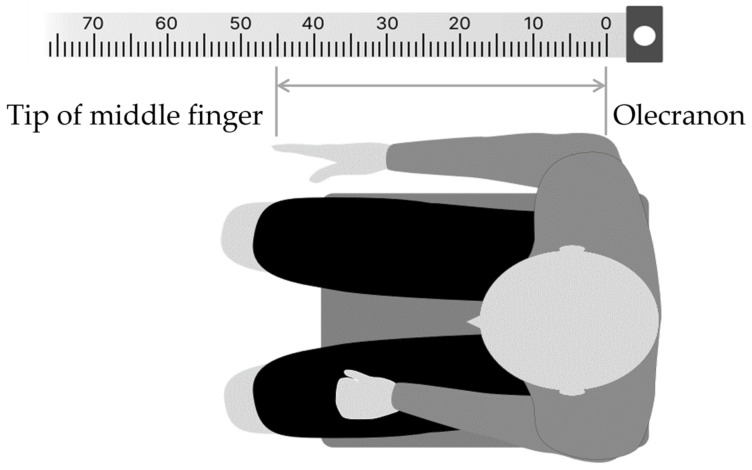
Forearm bisection task (FBT) procedure. The numbers at the top represent the paper ruler (cm) used to calculate the subjective midpoints.

In the FBT, the body image of the right forearm was assessed by pointing the left index finger to the midpoint of the right forearm, which was stationary with eyes closed. The method was as follows: first, in a resting chair position, the participant was asked to sit in a resting state for 5 min to control body perception by visual information immediately before the experiment, by covering both upper limbs with a towel to visually block them, while looking vaguely at a white wall (distance) in front of their eyes. The participants were then instructed to close their eyes, place their right forearm on the desk, and point to the midpoint of the forearm with the two ends of the elbow head and the tip of the middle finger (Figure 2). To prevent tactile feedback during pointing, a parallelepiped (70 × 10 × 11 cm) was placed over the test forearm. For a simple measurement of the subjective midpoint position, ruled tape with a scale index was attached to the top of the parallelepiped, and 0 cm was aligned with the elbow head. The percentage score of the subjective midpoint (FBT value) for each participant was calculated using Formula (1):

(1) $$FBT\;\mathrm{value}\;=\;\frac{\mathrm{Length}\;\mathrm{from}\;\mathrm{the}\;\mathrm{elbow}\;\mathrm{head}\;\mathrm{to}\;\mathrm{the}\;\mathrm{subjective}\;\mathrm{midpoint}}{\mathrm{Length}\;\mathrm{from}\;\mathrm{elbow}\;\mathrm{head}\;\mathrm{to}\;\mathrm{middle}\;\mathrm{finger}}\;\times \;100$$

No modifications were allowed during the task, and the pointing task was performed three times, with a washout period of approximately 5 min between sessions.

### 2.5. Body Cognition Assessment System (BCAS)

To assess the potential body cognition of the upper limb in real-time, a novel BCAS tool was developed that can directly assess body movement images using a relatively easy-to-imagine movement in the original movement of the upper limb function (in this study, raising the upper limb forward) as the assessment task.

**Figure 3:**
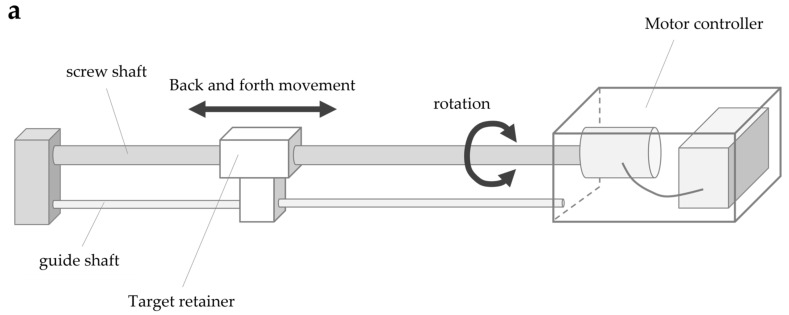

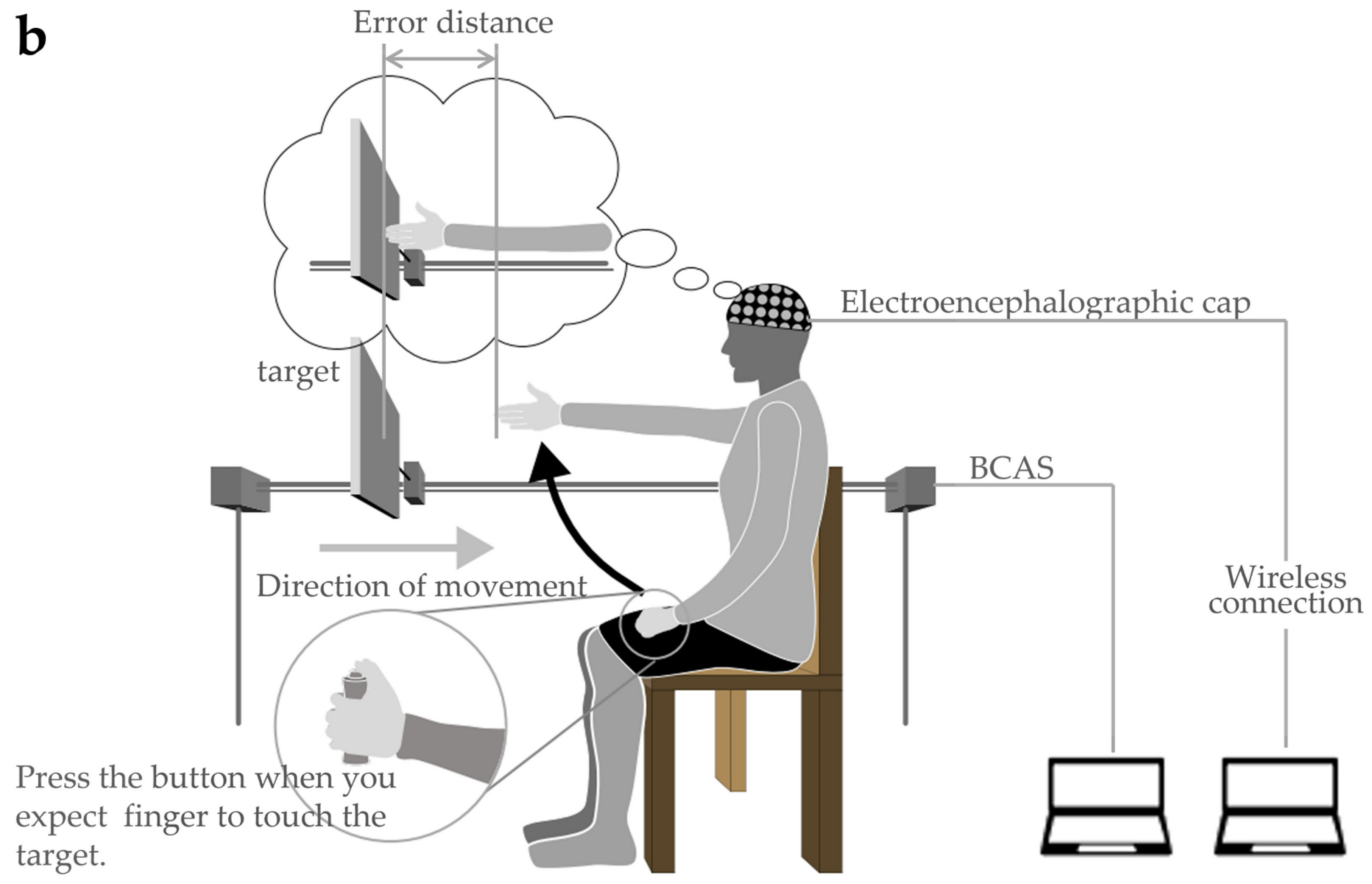
Body cognition assessment system (BCAS): (**a**) BCAS system; (**b**) BCAS measurement scene.

The system consisted of four parts: a screw shaft, guide shaft, target retainer, and motor controller (Figure 3a). The screw shaft was connected to the motor, and the target retainer was connected to the screw and guide shafts. The rotary motion of the screw shaft caused by the motor caused the target retainer to move horizontally along the shaft axis. The target retainer was moved at a constant speed by controlling the motor speed, consisting of five steps: 4, 7, 9, 11, and 14 mm/s. The motor speed was controlled by the number of pulse signal inputs to the motor (steps/s) (hereafter referred to as pulse speed) according to the selected speed. The movement of the target retainer was calculated using the following formula, which is based on the pulse speed, pitch of the screw shaft (15 mm), and number of pulse signals per motor revolution (one revolution in 800 steps for this motor): Equation (2)

(2) $$\;\mathrm{The}\;\mathrm{amount}\;\mathrm{of}\;\mathrm{movement}\;\mathrm{of}\;\mathrm{the}\;\mathrm{target}\;\mathrm{retainer}\;[mm]=\frac{15[\mathrm{mm}]\times \mathrm{pulse}\;\mathrm{speed}\;[\mathrm{step}/\sec]}{800[\mathrm{step}]\times \mathrm{travel}\;\mathrm{time}\;[\sec]}$$

The target retainer travel was added during forward motor rotation and subtracted during reverse rotation.

In the evaluation, the target board approaching from the front of the participant was stopped when the participant judged it to be approaching his own upper limb reach distance. The error between the visually measured reach distance and the actual reach distance was measured, and the size of the error was analyzed as the body cognitive ability value (hereinafter referred to as the BCAS value). The specific evaluation method is described as follows: The method involves fixing the trunk to the back of a chair in a resting chair position to prevent forward leaning of the trunk during the upper limb’s reaching movement. In addition, as in the FBT, both upper limbs were visually blocked by covering them with towels (to control body perception using visual information immediately before the experiment). The participants were allowed to remain in a resting state for 5 min while gazing blankly at a white wall (distance) in front of them. In the actual measurement situation (Figure 3b), the device was placed on the parallel bars used for gait training (GH-2600 standard mobile parallel bars, OG GIKEN Co., Ltd., Okayama, Japan) in a calm environment and fixed with a belt. Next, the participants were instructed to visualize their arms hanging vertically along their trunk, straight out in front (shoulder joint flexion 90°, internal/external rotation 0°, internal/external rotation 0°) against a target board (40 cm long × 25 cm wide) approaching at a constant speed, and press the button with their left thumb. When the fingertip touched the approaching target plate, the left thumb was used to press the button switch, and the target was stopped simultaneously. The right upper limb was then raised and the error distance from the stopped target board was measured. The error distance was considered a positive value if it was estimated to be shorter than the actual upper limb length and a negative value if it was estimated to be longer. In addition, the BCAS value was calculated using Equation (3), as it could be influenced by the participant’s actual upper limb length.

(3) $$BCAS\;\mathrm{value}\;=\;\frac{\left(\mathrm{upper}\;\mathrm{limb}\;\mathrm{length}\;-\mathrm{error}\;\mathrm{distance}\right)}{\mathrm{upper}\;\mathrm{limb}\;\mathrm{length}}\times 100$$

The movement of the target and calculation of the distance was performed using a controller connected to a measuring device. The tests were performed in triplicates. Between each measurement, a washout period of approximately 5 min was allowed, with controlled movement of both the upper limbs. The actual upper limb length was measured from the acromion to the distal end of the hand at 90° flexion of the shoulder joint, full extension of the elbow joint, and mid-forearm rotation/extension in the sitting position (on the resting chair).

### 2.6. EEG Measurement

EEG measurements were performed to compare the neural activity of the brain regions that create body cognition during the FBT and BCAS and to evaluate whether the tool is useful as a new assessment method for body cognition.

Polymate V (AP5148; Miyuki Giken Co., Ltd., Tokyo, Japan) and active dry electrodes (Miyuki Giken Co., Ltd.) were used to measure the EEG signals. Earth electrodes were then placed on the left earlobe. An external input cable was used to connect the BCAS to the EEG and synchronize the trigger to start recording. In addition, EEG was recorded in 28 channels (Fpz, Fz, Cz, Pz, Oz, Fp1, Fp2, F7, F8, F3, F4, C3, C4, C5, C6, P3, P4, P7, P8, O1, O2, T7, T8, and CPz), and a reference electrode was also placed in the left ear lobe. The sampling rate was 1000 Hz. The bandpass filter was 0.5–30 Hz. EEG signals were recorded during the interval between when the target started approaching the participant and when the switch was pressed to stop the target.

### 2.7. Data Analysis

For the FBT and BCAS values, Shapiro–Wilk test was performed to evaluate the normality of each dataset. The intra-class correlation coefficient (ICC) was obtained to verify the reproducibility of the results for each implementation time. In case the results exhibited variations from one evaluation to another, a one-way analysis of variance (ANOVA) was used to determine the significant differences between each of the three implementations.

For brain neural activity during the FBT and BCAS by EEG, the EEG data were analyzed by independent component analysis (ICA), using electromagnetic source estimation (Cortech Solutions, Inc., Wilmington, NC, USA) to eliminate noise, and by EEG imaging filter exact low-resolution brain electromagnetic tomography analysis (eLORETA). Cortical current density distributions were reconstructed from the denoised and normalized EEG data. The eLORETA analysis was performed using the Montreal Neurological Institute (MNI) 152 template built into the filter program and superimposed onto the stochastic anatomical template, which was superimposed post-drawing. The criterion for determining predominant neural activity was the average values of the brain neural activity (µA/mm2) for the first three datasets. Additionally, the two standard deviations were calculated, and the regions with neural activity above the threshold were calculated and identified as predominantly active regions. The relevance of these neural brain activities in the neural basis of body perception was investigated.

The relationship between the BCAS and each assessment was then verified by calculating Spearman’s rank correlation coefficient for the correlation between the KVIQ and BCAS values and Pearson’s product rate correlation coefficient for the correlation between the FBT values and body values. SPSS Statistics (version 22.0; IBM Japan) was used for the analyses, with a statistical significance level of less than 5%. The effect size was calculated using the G*Power ver. 3.1.9.6 [40]. G*power was set as follows: test family, *t*-test; statistical test, correlation; error probability, 0.05; and the effect size was calculated from the respective correlation coefficients.

## 3. Results

### 3.1. Reproducibility of Each Test

**Figure 4:**
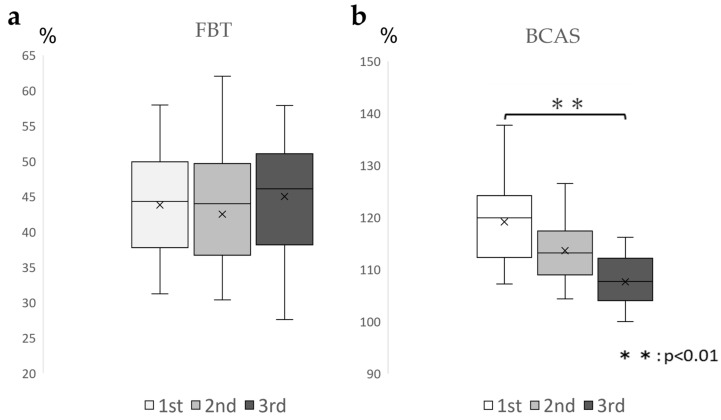
Changes in the FBT and BCAS values in three enforcements (**a**): Box-and-whisker diagram of the FBT values for each of the three implementations, displaying FBT values that were ICC (1.3) = 0.828 (0.609–0.935), above 0.7, indicating high reproducibility. (**b**): Box-and-whisker diagram of the BCAS values for each of the three exercises, showing that the BCAS values decreased rather than remained constant from session to session with ICC (1.3) = 0.372 (−0.513–0.78). FBT, forearm bisection test; BCAS: body cognition assessment system; ICC: intraclass correlation coefficient.

For the FBT values, the ICC (1.3) was 0.828 (0.609–0.935), which was above 0.7 and highly reproducible; for the BCAS values, the ICC(1.3) was 0.372 (−0.513–0.78). The BCAS values varied from session to session (Table 2). Based on the aforementioned results, one-way ANOVA was conducted for the BCAS values. The results demonstrated a significant difference between the conditions (F = 9.86, *p* < 0.01), and the Bonferroni test demonstrated no significant difference between the first and second sessions or between the first and third sessions. (Figure 4). Many participants estimated their right upper limb lengths to be longer than their actual upper limb lengths.

### 3.2. EEG Analysis

#### 3.2.1. Areas of Brain Activity during FBT

**Figure 5:**
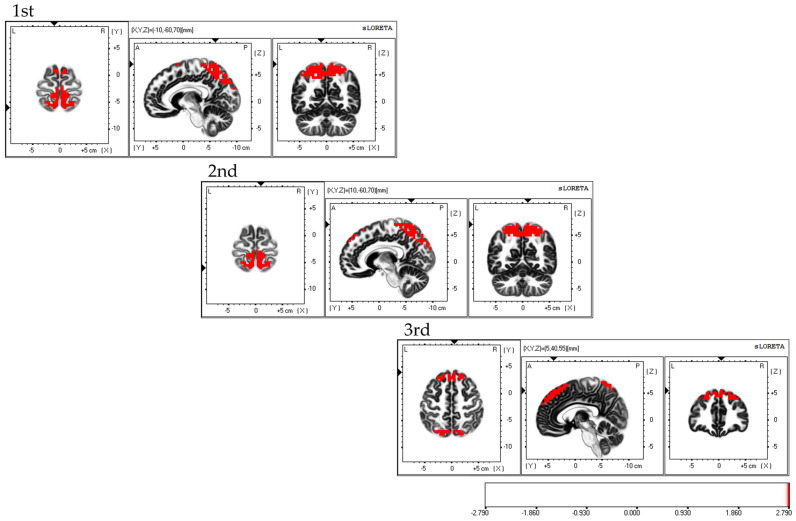
Neural activity during FBT. The results of the EEG analysis using eLORETA during the FBT. During FBT, parietal-lobe-dominant activity is observed, mainly in the bilateral superior parietal lobes during the first, second, and third sessions. Bilateral supplementary motor cortex activity is also observed during the three sessions. The eLORETA scale depicts brain regions with activity values above two standard deviations of the neural activity value (µV/mm^2^) as red regions. FBT: forearm bisection test; eLORETA: exact low-resolution brain electromagnetic tomography.

Considering the areas of brain activity during the FBT, the first and second sessions displayed parietal lobe predominance, mainly in the bilateral superior parietal lobes and activity in the bilateral supplementary motor areas, while the third session demonstrated a frontal lobe predominance, mainly in the dorsolateral prefrontal cortex and activity in the bilateral superior parietal lobes; the fourth session exhibited activity in the bilateral superior parietal lobes (Figure 5).

#### 3.2.2. Areas of Brain Activity during BCAS

**Figure 6:**
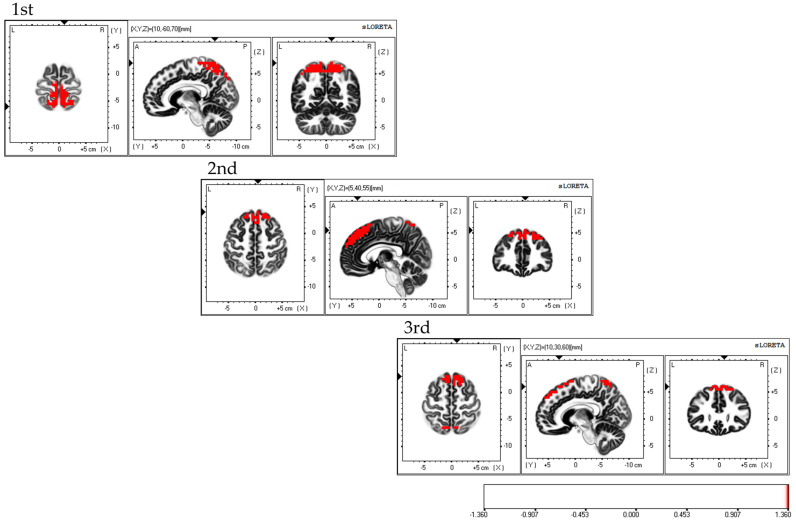
Neural activity during BCAS. The results of the EEG using eLORETA upon using the BCAS are as follows: the first session exhibited predominant neural activity mainly in the bilateral superior parietal lobes but also in the bilateral supplementary motor cortex and inferior parietal lobule; the second session demonstrated predominant neural activity mainly in the bilateral dorsolateral prefrontal cortex, along with the supplementary motor cortex and superior parietal lobule; the third session exhibited predominant neural activity in the bilateral supplementary motor cortex, along with the dorsolateral premotor cortex, dorsolateral prefrontal cortex, and superior parietal lobule. Variations in brain activity were observed after each session. The eLORETA scale depicts the brain regions with activity values above two standard deviations of the neural activity value (µV/mm^2^) in red. BCAS: body cognition assessment system; eLORETA: exact low-resolution brain electromagnetic tomography.

Considering the areas of cerebral neural activity during BCAS, the first session demonstrated predominant neural activity in the bilateral supplementary motor cortex and inferior parietal lobule, mainly in the bilateral superior parietal lobule. The second session demonstrated predominant neural activity in the supplementary motor cortex and superior parietal lobule, primarily in the bilateral dorsolateral prefrontal cortex. The third session demonstrated predominant neural activity in the bilateral supplementary motor cortex, with predominant neural activity in the dorsolateral premotor cortex, dorsolateral prefrontal cortex, and superior parietal lobules (Figure 6). Each of the three sessions exhibited fluctuations in the areas of brain activity.

### 3.3. Relevance of the Evaluation Indicators

As for the relevance of each assessment, significant correlations were identified between (1) KVIQ (5Kd mother-finger-tip) and BCAS values (3rd time) (r = 0.53) {power (1-βerror prob), 0.65}; no correlation was observed between the FBT values–BCAS values (2) KVIQ (5Kd mother-finger-tip) and BCAS values (3rd time) {power (1-βerror prob), 0.65}; no correlation between the FBT values–BCAS values and (3) KVIQ (5Kd mother-finger-tip) and BCAS values (3rd time) (r = 0.53) {power (1-βerror prob), 0.65}. A significant correlation was also observed between (2) the first and second BCAS values (r = 0.611) {power (1-βerror prob), 0.82} and (3) the second and third BCAS values (r = 0.741) {power (1-β error prob), 0.98} (Table 3).

## 4. Discussion

In the present study, we developed the assessment tool, the BCAS, and validated and compared it with existing assessment methods to investigate whether it could be used as a new method for assessing body cognition.

The BCAS values and brain activity during the assessment run demonstrated low reproducibility (ICC [1.3] = 0.372 [−0.513–0.78]); the BCAS values varied across the three runs, and brain activity exhibited changes in each run. The first session demonstrated activity mainly in the bilateral superior parietal lobes, as well as in the bilateral supplementary motor cortex and inferior parietal lobes. The supplementary motor cortex and parietal association cortex are areas that match and compare visual information about the target and surrounding spatial information with body movement images. Furthermore, they are responsible for preparatory activities for the onset of movement [13]. The parietal association cortex is considered an active base for body movement imagery and a sense of body ownership [41,42]. Activities in the parietal association cortex are involved in the induction of a sense of agency. The findings suggest that the initial assessment of the BCAS could create a sense of bodily possession and that the second assessment demonstrated activity in the dorsolateral prefrontal cortex, as well as in the supplementary motor cortex, and right superior parietal lobule. The dorsolateral prefrontal cortex and supplementary motor cortex are considered to be active during body movement imagery [43] and during the creation of a sense of ownership of action and are involved in action planning [44]. Furthermore, coupling (cooperative activity) between the supplementary motor cortex and dorsolateral prefrontal cortex is involved in motor imagery responses, which play an important role in the motor control required for upper limb movements [45]. Thus, the second BCAS assessed the creation of the state of action subjectivity [46] based on somatomotor imagery modified by the first experience, whereas the third BCAS demonstrated activity mainly in the bilateral supplementary motor cortex, dorsolateral prefrontal cortex, and superior parietal lobule, based on Spearman’s rank correlation coefficient results. A significant correlation was observed between the third BCAS value and the physical motor imagery assessment, KVIQ “5Kd Thumb-finger opposition”. Regarding brain activity during motor imagery, the primary motor, supplementary motor, and premotor cortical areas are well known, including areas related to action planning, such as the dorsolateral prefrontal cortex, inferior frontal cortex, and posterior parietal lobes [47]. Moreover, when the responsiveness of body movement images to actual movements increases, body movement images are formed from memories in the supplementary motor cortex [48], with the prefrontal cortex becoming more active through these brain networks [49]. A significant correlation was observed between the third BCAS value and the KVIQ “5Kd. mother finger-fingertip”. Considering the fact that the third session also captured a state reflecting the creation of body-motor imagery, including cognitive function activity, the BCAS values probably demonstrated fluctuations in the three executions; moreover, the one-way ANOVA displayed no significant difference between the first and second sessions but significant differences between the first and third sessions. In terms of brain neural activity during BCAS, changes were observed in each execution, with brain activity centered in the superior parietal lobule in the first session, the prefrontal cortex in the second, and supplementary motor cortex in the third session, suggesting that the above results reflect the creation of a sense of body ownership in the first session, a sense of action ownership in the second session, and a body-motor image in the third session. The senses of body and action ownership follow changes in the environment and change almost in real time; this is called the fast dynamics [50] of the motor learning process. Fast dynamics are active during the early stages of motor learning, mainly in the frontal and parietal regions, and are thought to play a predominant role in guiding long-term motor learning [51]. Based on EEG analysis, the results of the present BCAS values suggest that the condition reflects early motor learning and that a single assessment can lead to changes in body cognition. The one-way ANOVA demonstrated that there was no significant difference between the first and second evaluations, whereas a significant difference between the first and third evaluations was observed. Wen et al. [52] stated that the presence of a goal in hand movement and feedback on the achievement of that goal promoted the updating of the body-motor image through a sense of body ownership and motor subjectivity indication. In the present BCAS, after the target was stopped by pressing a switch, the participants were asked to perform a reaching movement after blocking the visual information to avoid updating the body perception of the upper limb through visual information and measuring the error distance. However, feedback on external information about the target not touching the fingertips was generated. However, feedback about the external information occurs when the target is not touching the fingertips. Therefore, for the third time, the body movement image was renewed through the first and second experiences. In conclusion, the results of the EEG analysis suggest that the fluctuations in the BCAS values with each enforcement of the program reflect initial motor learning and that the sense of body possession in the first session and sense of action ownership in the second session indicate preparatory activity for the third physical movement image.

Considering the FBT values and brain neural activity during the evaluation run, a high reproducibility of the FBT values was observed in both runs (ICC [1.3] = 0.828 [0.609–0.953]). Parietal dominance centered on the superior parietal lobule, and the supplementary motor cortex was active during the first and second runs. The activity in the superior parietal lobule was identified to be highly reproducible. Additionally, the superior parietal lobule is important for generating a sense of body possession and is essential for maintaining body image [43]. Furthermore, as the lobule uses past visuospatial information to determine and predict position and size [53], the area was considered to be predominantly active in the FBT, pointing to the midpoint of the right forearm with the eyes closed. As for the supplementary motor cortex, it is responsible for providing sensory results predicted before movement execution to the brain regions responsible for movement execution [54]. In the FBT, without actually touching the limb, the supplementary motor cortex may be activated based on the prediction mechanism of touch when a sense of ownership is created [55]. In the third session, the dorsolateral prefrontal cortex was mainly frontal and the superior parietal lobule was active. The dorsolateral prefrontal cortex is a region with working memory functions that are also involved in body image [21]. Working memory is the ability to temporarily store and manipulate information required for cognitive tasks, a property that allows one to respond flexibly to the demands of various activities and tasks [56,57]. The third time captured a state reflecting body image creation in the right forearm, which was modified during the first and second FBT sessions. In conclusion, the results of the present study support previous reports that the FBT is an evaluation index of body possession from a neurological perspective, and the small variation and high reproducibility in the three administrations of the FBT are attributed to the fact that the basis of body possession is formed by hetero-sensory integration, including vision and touch [58]. Dalila et al. [59] demonstrated that visual and tactile information is not always necessary for the creation of a sense of ownership. In the FBT conducted in the present study, the right forearm was covered by a parallelepiped, and fingering was performed with eyes closed; therefore, visual and tactile feedback was considered scarce. Therefore, it is suggested that a sense of body possession is formed in the superior parietal lobule from past visual information and the intrinsic sensory information of the right forearm at that time. [44,55] Furthermore, the environment was poor in sensory feedback related to the right forearm, which may have prevented the renewal of the sense of body ownership from occurring and resulted in less fluctuation in the FBT values.

The findings suggest that the BCAS may capture body perceptions that change immediately, whereas the FBT may assess the sense of body possession based on highly reproducible and static body images. Since body cognition changes constantly [17,18], BCAS may be useful in capturing immediate changes in body perception.

The fact that a relationship was observed between the first and second BCAS values and between the second and third BCAS values based on Pearson’s product-rate correlation coefficient suggests that the brain activity areas were identified by the BCAS in the study. The activity is responsible for the formation of body diagrams that are updated by integrating input information, such as visual, proprioceptive, and tactile inputs related to the body [60]. The fact that no correlation was observed between the BCAS and FBT values suggests that the BCAS is a tool for assessing dynamic body movement imagery in which the right upper limb is raised and held forward and that the FBT is a tool for assessing dynamic body movement imagery in which the right upper limb is raised and held forward. The FBT assesses static body image by pointing to the midpoint of the right forearm on a desk [25,26,27]. Therefore, functional differences may exist between dynamic and static imagery.

The limitations of the present study include the fact that only the myosensory imagery scale was measured in the KVIQ, a first-person body movement imagery assessment, and not the visual imagery scale. The newly developed BCAS may have created body awareness by using visual information on the target board as a cue and matching it with the body movement image of the right upper limb’s reaching movement. As the assessment probably included elements of visual information and images of the right upper limb, the BCAS and KVIQ visual imagery scales may have been more conducive to correlations. Second, the BCAS was not subjected to brain network analysis, and the association between the brain regions that showed predominant activity during each administration could not be objectively assessed. Therefore, the results were considered unreliable. In recent years, several neuroimaging studies have investigated the neurofunctional correlates of the sense of body ownership and action subjectivity. Body cognition occurs through interactions between the frontal and parietal lobes and the insular cortex, which form a network [61,62]. Therefore, the brain networks involved in somatocognition should be examined in detail in future studies. Third, the number of subjects in this study was 16, which was insufficient to prove the usefulness of BCAS more precisely. Therefore, in future studies, the number of subjects should be increased, and the BCAS should be validated from multiple perspectives by comparing it with body cognitive assessments other than the FBT and KVIQ.

## 5. Conclusions

In this study, the reproducibility and usefulness of the newly developed BCAS were tested by comparison with the established KVIQ and FBT assessments. The results suggest that the BCAS can objectively capture immediate changes in somatocognition and can potentially be used as an assessment method. In the future, conducting detailed validation of the usefulness of this assessment method for patients with sensorimotor dysfunction, due to stroke or spinal cord injury, which foregrounds the impairment of body cognition, is warranted.

## Figures and Tables

**Table 1:** Demographic characteristics of the participants.

Characteristics	Participants (*n* = 16)
Gender	All male
Age	21.2 ± 1.6
Dominant hand	All right
Upper limb length (Right)	729.7 ± 25.6 (mm)

**Table 2:** Measured FBT and BCAS values and ICC (*n* = 16).

	Mean (SD)			Intraclass Correlation Coefficients (ICC)			
	1st	2nd	3rd	ICC (1.1)	95% CI	ICC (1.3)	95% CI
FBT value (%)	43.8 (7.6)	42.5 (10.5)	45.0 (8.2)	0.616	0.342~0.827	0.828	0.609~0.935
BCAS value (%)	119.2 (8.4)	113.6 (6.4)	109.1 (7.2)	0.165	−0.127~0.542	0.372	−0.513~0.78

FBT: forearm bisection test; BCAS: body cognition assessment system; ICC: intraclass correlation coefficients.

**Table 3:** Correlation between BCAS values and each assessment from the first to the third session.

	BCAS Value (1st)		BCAS Value (2nd)		BCAS Value (3rd)	
	rs	*p*	rs	*p*	rs	*p*
BCAS (2nd)	0.611 *	0.016			0.741 *	0.002
FBT value (average of 3 times)	0.179	0.524	−0.004	0.990	0.222	0.446
KVIQ 3knd forward shoulder flexion	−0.009	0.973	0.008	0.979	0.161	0.584
KVIQ 4Kd elbow flexion/extension	−0.295	0.286	−0.146	0.604	0.093	0.751
KVIQ 5Kd thumb-fingers opposition	0.013	0.963	0.093	0.743	0.534 *	0.049

* *p* < 0.05; BCAS: body cognition assessment system; FBT: forearm bisection test; KVIQ: The Kinesthetic and Visual Imagery Questionnaire.

## Data Availability

Data supporting the results of the study are available from the corresponding author, T.K., upon reasonable request.
